# Exophthalmic Goitre, or Graves’ Disease

**Published:** 1886-01-20

**Authors:** J. W. Thompson

**Affiliations:** St. Paul, Minn.


### EXOPHTHALMIC GOITRE, OR GRAVES' DISEASE.

By J. W. Thompson, M. D., of St. Paul, Minn.

During the past ten years that I have been engaged in the practice of ophthalmic medicine and surgery, I have had the opportunity of seeing a considerable number of cases of exophthalmic goitre. The pathogeny and treatment of this remarkable disease are very unsettled, and therefore very unsatisfactory. With a view of adding something to my limited knowledge of this subject beyond what I was able to gather from its literature, I adopted, in the beginning, the practice of keeping somewhat extended notes of every case of the disease that came under my observation. I have selected five typical cases for the subject of this paper, and herewith present the results of my observations to the readers of the Journal.

Palpitation of the heart, enlargement of the thyroid gland, and prominence of the eyeballs, formed a marked trio of symptoms which were present in all five of the cases selected. Each one of these great symptoms occurred in point of time in the order above mentioned. The length of time between the occurrence of these symptoms in their regular order was not constant The longer the interval between the heart disturbance and the enlargement of the thyroid gland, the more unyielding was the malady to remedial agents, and the more protracted and intense was the suffering of the patient. One of the five cases occurred in a male thirty years of age. Of the four females, the disease occurred in one before the menopause, and in the other three at or about that period. The heart palpitation in the male was very distressing several weeks before the other symptoms made their appearance. In his case the enlargement of the thyroid was very slight, while the protrusion of the eyeballs was very considerable. In all other respects he was healthy and robust, and had always enjoyed good health. There seemed to be nothing in his individual history that furnished any apparent cause or foundation for the disease. Upon investigating his family history, he stated that one member of his family—a brother—had in early youth suffered with "fits," as he termed it. A careful inquiry revealed the fact that the "fits" had undoubtedly been epilepsy. Possibly this peculiar neurosis manifested itself in the one by epilepsy and in the other by exophtalmic goitre.

Of the four cases in females—in three of them, the three characteristic symptoms exophthalmos, goitre and quickening of the heart's action, were well marked. In the fourth one the enlargement of the thyroid was confined to the left lobe. Over the right lobe only a very trifling fulness could be detected. The prominence of the eyeballs was very great. At times, the insertion of the recti muscles could be seen. The eyes became very sensitive to the ight, and, in one, a central corneal formed, which is said by some authors never to occur. The severity of the symptoms was so intense that the patient was confined to her bed the greater part of the time for a period of nearly four months. Under appropriate treatment, the ulcer healed kindly, the patient made a good recovery, and four years afterwards was enjoying good health. It is over a year since I lost sight of her, and I am unable to say whether there has been any recurrence of the disease.

The general symptoms in all the cases, except the male, were very like. Great excitability, anæmia and emaciation were present in a very marked degree. The rapid and forcible action of the heart produced a rapid, jerky, trip-hammer pulse. The carotids throbbed perceptibly, and the pulsation of the abdominal aorta could be very distinctly felt. In one of the cases I found the pulse as frequent as 170 per minute. In none of the cases did I find it below 100. There existed in all an extremely intensified self-consciousness. The most trivial exciting cause produced flushing of the face, great increase of the heart's actioo, and occasionally much dyspnœa, which, in one case, became at times rather alarming. In this case, however, some of the dyspnœa could be readily attributed to the excessive enlargement of the thyroid. The enlarged thyroid increased the size of the neck to such a degree, that the collar she formerly wore was too small by four and one-half inches.

Among the general symptoms, besides the palpitation of the heart, goitre and exophthalmos, there were a number of minor symptoms which were more or less present in all the cases, and proved, at times, annoying and even distressing. These were indigestion, hepatic disorder, pains in the extremities, local dropsy, sleeplessness, headache, vertigo, ringing in the ears, nausea, and vomiting after meals, hiarrhœa, hæmorrhage from the bowels, nose-bleed, night-sweats, and, in two of the cases, hæmoptysis. There was also much irritability of temper. The mind became at times very capricious and suspicious, feeble and childish.

One of the prevailing theories of the day is that this peculiar disease has its origin in the nerves of organic life. From the observations that I have had the opportunity of making, I have become a little skeptical on this point. It seems to have been established more by assertion than by proof. With a view of throwing some light, if possible, on the correctness or incorrectness of this theory, I made a very careful record of the temperature at stated times in each and every case that cama under my observation. In each instance I found it above the normal. It ranged from 99 to 102. It was usually increased when, from any exciting cause, the action of the heart was accelerated. The temperature was always taken on each side of the body at the same time, and it always registered the same; *e.g.* the corresponding cheeks, the sides of the neck, and the axilla. The normal difference between the cheeks and the axilla was observed in each insrance, wlth one or two slight exceptions hardly worthy of mention. It has been quite conclusively shown by experiment that the ganglionic chains have an action entirely independent of each other. If the cervical portion of the ganglionic system be involved, as is claimed, then there would necessarily exist a difference of temperature between the oorresponding sides, since it is impossible for both chains to be affected equally in the outset and progress of every case of this disease.

Another observation made in relation to thc five cases under consideration, that opposes the sympathetic theory, was the prompt response of the iris to the action of light and shade, and to atropine. The writer does not claim that the phenomena furnished by five typical cases alone could possibly establish a correct theory of the origin of a disease, which furnishes such a multitude of symptoms having such a wide range of deviation. He does claim, however, that the sympathetic theory is quite inadequate to explain all the phenomena observed in these cases.

Dr. Hammond says, in his celebrated work on "Diseases of the Nervous System," page 819, seventh edition: "I am inclined to think that in the present state of our knowledge we are scarcely warranted in locating exophthalmic goitre in the sympathetic nervous system, and that we are justified in regarding it as an affection of the brain and medulla oblongata."

The link that connects this disease to ophthalmology is the local effect it produces upon the eyeballs. This is, however, only one of its pathognomonic symptoms. Yet, it is a symptom that produces a hideous deformity and has given much notoriety to the disease. It gives to the eyes a wild, staring expression. They project forwards from the orbits, in many instances, so far that the lids cannot close over them entirely. The prominence of the globe became so marked in one case that the lids at no time, not even while sleeping, completely covered the cornea. A very interesting case of this nature is given in Trosseau's "Chemical Medicine," vol. 2, p. 178. He says: "Dr. Pain kindly supplied me with further details of this interesting case. Twice in the course of a year there came on such a paroxysmal increase of the exophthalmos, that one of the eyeballs became dislocated as it were. . . . The eyelids got behind the circumference of the eyeball, which had to be pushed back with a certain amount of force in order to get the lids to come forwards again." This prominence of the eyeballs is due largely to hyperæmia of the orbital cushions, and, in the chronic cases, there is undoubtedly some hypertrophy. Muller's muscle, which has the power of projecting the eye forwards in health, is supposed by some to act as one of the projecting factors in this deformity. In these extreme cases of prominence, the cornea is constantly exposed to the irritating action of the atmosphere and dust. Occasionally, in very chronic cases, the conjunctiva of the upper lids becomes much thickened and the surface of the cornea uneven and vascular.

In most of the cases that I have been able to observe carefully, the prominence of both eyes was about equal, and, as nearly as I could ascertain, occurred simultaneously. In many of the cases, however, the extent of the protrusion was rather deceptive. The eyelids seemed in some instances to become retracted towards the orbit and favored, as it were, their lodgment behind the equator of the eyeball. It requires but a small increase in the width of the sclerotic zone to give to the eye the appearance of more prominence than in reality exists. In one case I observed a considerable degree of anæsthesia of the cornea, so that the finger could be passed over it gently without producing but slight discomfort to the patient. I believe this is the exception, and not the rule. In the cases of extreme prominence, I found hypermetropia in a much higher degree than I had reason to believe existed before the occurrence of the exophthalmos. This was the mechanical result produced by the enlargement of the orbital cushions, which pressed the globe forwards against the resisting lids, and thus resulted a shortening of the antero-posterior axis of the globe.

The prognosis in this disease is usually favorable, though, when required to express an opinion with reference to any particular case, it is prudent to be guarded, since cases have been known to terminate fatally. I never saw but one case having a fatal termination. In certain mountainous regions in France, this disease has been known to prevail somewhat as an endemic, and occasionally one has had a fatal termination. When death does occur, it is usually the result of general exhaustion and a gradual failure of all the vital forces. The victim is by degrees worn out.

Tke treatment is about as unsettled as the etiology. The materia medica has been pretty thoroughly ransacked, without avail, for a specific. At one time and another I have employed a variety of treatment. Iodine exhibited externally and internally seemed to be attended with more favorable results, as far as my experience has gone, than any other medicinal agent. Yet, in some of the cases, the hygenic and diateric regulations, together with a change of climate when the circumstances of the patient permitted, had undoubtedly a favorable influence in bringing about a favorable result.

I think the only safe plan of treatment is to endeavor to guide the malady to a favorable end. To attempt to cut it short by specific measures, seems too much like striking in the dark. Many times I believe it is difficult to say whether the patient is cured or got well.—*Jour. American Med. Association.*

